# Neural correlates of somatoform disorders from a meta-analytic perspective on neuroimaging studies

**DOI:** 10.1016/j.nicl.2016.04.001

**Published:** 2016-04-10

**Authors:** Markus Boeckle, Marlene Schrimpf, Gregor Liegl, Christoph Pieh

**Affiliations:** a Department of Psychotherapy and Biopsychosocial Health, Donau-Universität Krems, Krems, Austria; b Medical Clinic, Department of Psychosomatic Medicine, Charité—Universitätsmedizin, Berlin, Germany; c Karl Landsteiner University of Health Sciences, Krems, Austria; d Department of Psychosomatic Medicine, University Hospital Regensburg, Regensburg, Germany

**Keywords:** Somatoform disorders, Somatoform pain disorders, Neuroimaging, MRI, ALE, Activation likelihood estimation, Meta-analysis, Premotor area, Supplementary motor cortex, Middle frontal gyrus, Anterior cingulate cortex, Insula, Posterior cingulate cortex

## Abstract

Somatoform disorders (SD) are common medical disorders with prevalence rates between 3.5% and 18.4%, depending on country and medical setting. SD as outlined in the ICD-10 exhibits various biological, social, and psychological pathogenic factors. Little is known about the neural correlates of SD. The aims of this meta-analysis are to identify neuronal areas that are involved in SD and consistently differ between patients and healthy controls. We conducted a systematic literature research on neuroimaging studies of SD. Ten out of 686 studies fulfilled the inclusion criteria and were analyzed using activation likelihood estimation. Five neuronal areas differ between patients with SD and healthy controls namely the premotor and supplementary motor cortexes, the middle frontal gyrus, the anterior cingulate cortex, the insula, and the posterior cingulate cortex. These areas seem to have a particular importance for the occurrence of SD. Out of the ten studies two did not contribute to any of the clusters. Our results seem to largely overlap with the circuit network model of somatosensory amplification for SD. It is conceivable that functional disorders, independent of the clinical impression, show similar neurobiological processes. While overlaps do occur it is necessary to understand single functional somatic syndromes and their aetiology for future research, terminology, and treatment guidelines.

## Highlights

• Five neurobiological areas correlate with the occurrence of somatoform disorders (SD).
• Areas are crucial for emotional, evaluative and cognitive aspects of pain processing.
• Prefrontal areas indicate involvement of pain memory in SD.
• Functional disorders might have central sensitization as an underlying factor.
• Results do not support the classification of SD as medically unexplained symptoms.

## Graphical abstract

**Figure:**
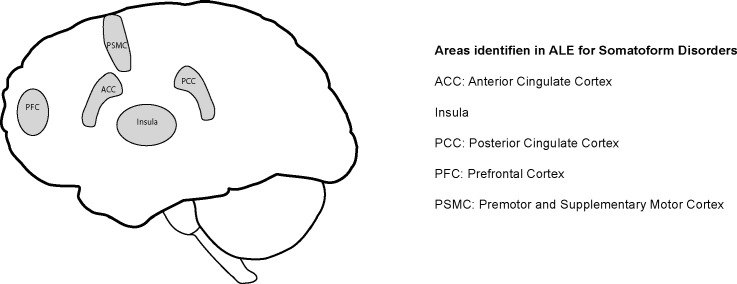

## 1 Introduction

Somatoform disorders (SD) are highly prevalent in many medical settings. In German psychosomatic hospitals, 18.4% of the inpatients fulfilled the criteria of the ICD-10 (WHO, 1992) for SD (Pieh et al., 2010). It is estimated that SD have a prevalence of 16.1% in primary care settings in the Netherlands (de Waal et al., 2004), a life-time-prevalence of 12.9% in Germany (Meyer et al., 2000) and a 12-month prevalence of 6.3% (Wittchen et al., 2011) in Germany. General practitioners overestimate the prevalence of patients with SD in their medical practice considerably with 27.7% (Boeckle et al., 2014). Because of high medical costs and indirect costs, such as those caused by times of un-employability and early retirement, patients with somatoform disorder generate high economic costs for the health system (Rief and Henningsen, 2011). Patients with somatoform disorders (F45.-) have physical symptoms that suggest a medical condition but are not or not fully explained by any other medical condition and are related to psychological factors (WHO, 1992). The symptoms must cause impairment in occupational, social or other areas, or clinically relevant stress (WHO, 1992).

While many potential pathogenic factors have been discussed for somatoform disorders (SD) and related diagnoses, the pathogenesis of the symptoms is still unclear. There is some evidence that physically and sexually abused people have a higher risk of developing a somatoform disorder (Paras et al., 2009), as are persons with insecure or disorganized/disoriented attachment style (Waller et al., 2004). Barsky (1992) describes a cognitive style called “somatosensory amplification”, which can be applied to many patients with somatoform disorder. Patients with somatoform disorder often exhibit a heightened focus on their own bodies, perceiving their bodily complaints quicker as illness than healthy people do. The term “central sensitization” has recently been used to describe a neurobiological process, which assumes that symptom onset is associated with a hyper-responsive neural network in high-risk individuals (Bourke et al., 2015, Nijs et al., 2012, Phillips and Clauw, 2011). Patients with SD rate normally innocuous stimuli as painful stimulation due to an alteration of the network (Bourke et al., 2015). Additionally, patients perceive their complaints as illness and thus display augmented bodily attention (Barsky, 1992). Risk factors for SD are, amongst others, personality traits like neuroticism and alexithymia, childhood adversity, physical trauma, infections as triggers, and changes in the hypothalamic–pituitary–adrenal axis or in the immune system (American-Psychiatric-Association, 2013, Bourke et al., 2015, Burba et al., 2006, Rief and Henningsen, 2011).

Neuroimaging methods, such as functional magnetic resonance imaging (fMRI), positron emission tomography (PET) and voxel-based morphometry (VBM) enable us to investigate neuronal activities and structural differences between different subpopulations. For instance, Yildirim et al. (2012) found smaller pituitary volumes in somatization patients than in a healthy control group, while Atmaca et al. (2011) examined differences in hippocampal and amygdalar volumes. Bourke et al. (2015) report that the most commonly found components of the neural network in central sensitization are, on the one hand the insula, which is involved in multimodal pain input (Peyron et al., 2002), threat detection (Critchley et al., 2002), interception (Craig, 2003a) emotional regulation (Gray et al., 2007), and motivation (Balleine and Dickinson, 2000), and on the other hand, the striatum (Zink et al., 2003), functioning in salience detection (Itti and Koch, 2001) in relation to onset speed (Iannetti et al., 2006), novelty (Iannetti et al., 2008), and context (Näätänen et al., 2007). Although neuroimaging findings have led to a better understanding of the pathogenesis of SD and other functional somatic syndromes (FSS), the evidence of neurobiological changes of FSS is still contradictory, as is true for SD. Additionally, there is evidence to support that FSS might be based on changes of the central nervous system, resulting in a common central augmentation of innocuous stimuli to pain (Bourke et al., 2015). The aims of this meta-analysis are to identify consistently differing neuronal areas that are involved in SD. Additionally, we hypothesize that identified areas coincide with theoretical models, e.g. central sensitization (Bourke et al., 2015, Perez et al., 2015).

## 2 Material and methods

### 2.1 Literature search

We searched the scientific databases Pubmed, ISI web of knowledge, Scopus, Cochrane database, Psycinfo, and Psyndex for relevant publications with the following terms: (“somatic symptom disorder” OR “somatoform disorder” OR “somatization” OR “functional somatic symptoms” OR “functional somatic syndrome” OR “somatization disorder”) AND (“neuro imaging” OR (“magnetic resonance imaging” OR (“magnetic” AND “resonance” AND “imaging”) OR “magnetic resonance imaging” OR “fmri”) OR (“magnetic resonance imaging” OR (“magnetic” AND “resonance” AND “imaging”) OR “magnetic resonance imaging” OR “mri”) OR PET OR VBM). Abstracts and titles were scanned by MB and CP, according to the a priori defined criteria (English publication; human adult subjects; no reviews, case reports, letters to editors or editorials: only original research; imaging methods PET, MRI, SPECT; including coordinates in Talairach space or MNI; differences between healthy subjects and patients; SD). Publications were included in the full text review if one of the raters found no exclusion criterion, resulting in a conservative approach and preventing the exclusion of possible full texts. We adopted the guidelines of the “PRISMA Statement” to provide transparent data selection (Moher et al., 2009). Only studies on somatoform disorder according to ICD or DSM matching ICD criteria were included. Although other pain related functional disorders, such as irritable bowel syndrome (IBS) or fibromyalgia syndrome (FMS) have partially similar diagnostic criteria (Eich et al., 2008). We specifically excluded IBS and FMS even though they are often described as syndromes sharing specific aspects (Häuser and Henningsen, 2014, Henningsen et al., 2007) while it is repeatedly emphasized that these syndromes are not the same (Bourke et al., 2015, Häuser and Henningsen, 2014, Lacourt et al., 2013, Rief and Isaac, 2014, White, 2010, White, 2013). Even though there are arguments for lumping FSS together as well as arguments for separate syndromes, we decided to base our selection criteria for the meta-analysis to a clear diagnosis in order to enhance interpretation of our results.

### 2.2 Meta-analysis

All coordinates from eligible publications were analyzed with an Activation Likelihood Estimation (ALE) using a Java-Version of GingerALE 2.3.5 (Eickhoff et al., 2009, Eickhoff et al., 2012, Turkeltaub et al., 2012). ALE is a quantitative and meta-analytical approach to calculate consistent differences in gray matter volume and/or activation, based on reported statistical differences in the literature. ALE is possible to conduct on structural as well as functional neuroimaging studies solely as well as in combination of structural and functional neuroimaging studies. We calculated two analysis, one including functional and structural studies, another focusing on functional studies. A subcategory analysis for structural imaging modality or certain paradigm was not feasible due to sample size. We included all coordinates that presented relevant differences between healthy controls and patients into our analysis.

All coordinates reported in MNI were recalculated into Talairach space via the icbm2tal tool (Laird et al., 2010, Lancaster et al., 2007) provided by brainmap.org (Eickhoff, 2014). We calculated a cluster-level of 0.05 with 1000 threshold permutations, a minimum cluster size of 240 mm^3^ and Cluster forming value of 0.001. ALE maps were visualized with Mango version 3.7 (1415) (Mango, 2014) to investigate threshold maps that were superimposed on a standard anatomical image (Colin.1.1.1.nii).

We summed healthy controls and patients to quantify our number of subjects. To calculate the most conservative ALE analysis, for each dataset we entered the number of individuals participating in the experiment with the lowest number of participants. Publications analyzing the same individuals were subsumed under one publication, in order to prevent overestimating the influence of these individuals on the whole sample. All reported foci of these publications entered ALE analysis, including the foci lying outside of the mask, as small proportions of outside foci do not influence results. In the results, the nearest gray matters within ± 5 mm are reported.

## 3 Results

### 3.1 Study selection

**Fig. 1:**
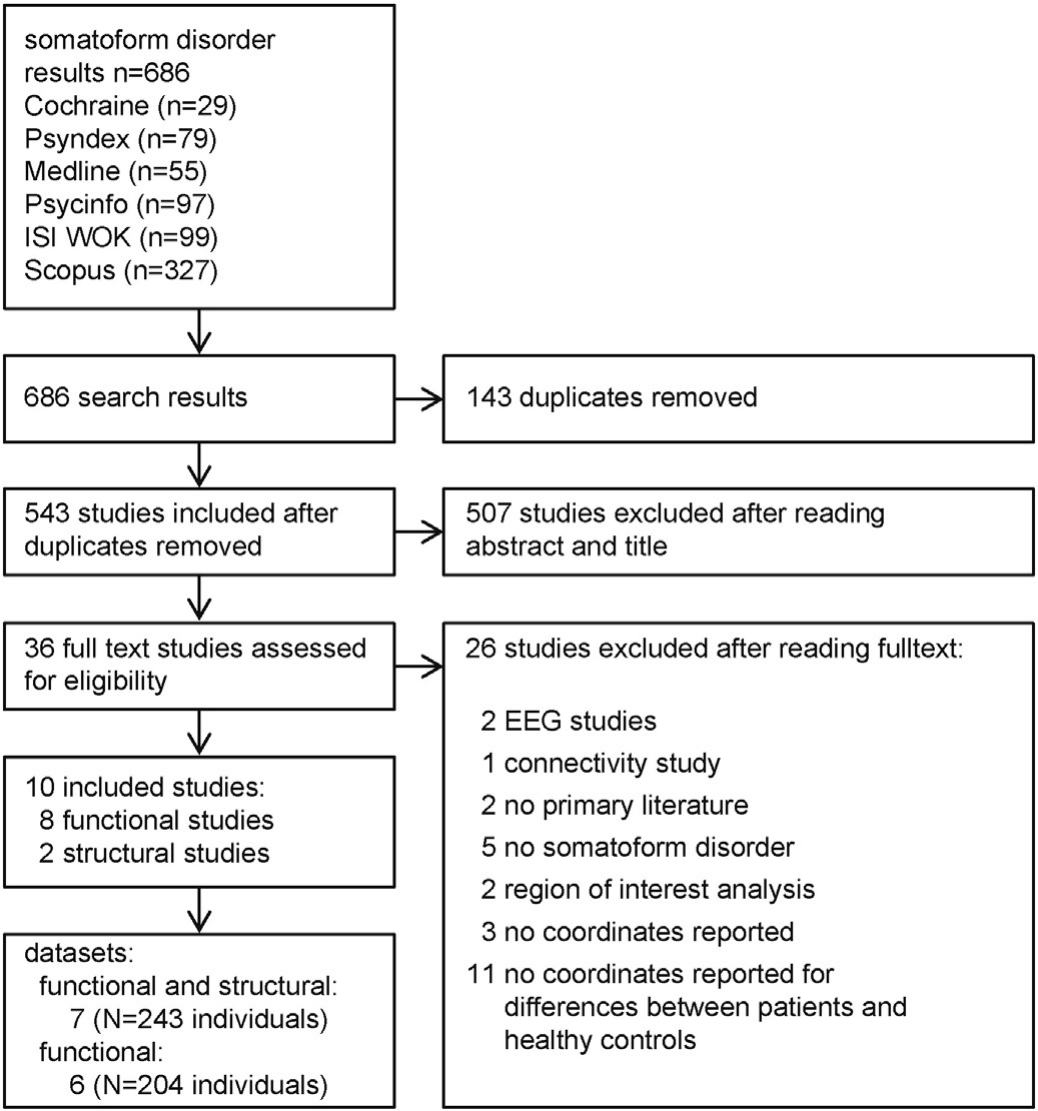
Flow chart of review process. Number of publications (n) and number of individuals (N) are indicated.

The systematic literature search yielded 686 research articles on SD (Fig. 1). Search results included 143 duplicates, resulting in 543 studies eligible for abstract and title review. The title and abstract review resulted in 507 excluded studies, leaving 36 studies for full text analysis. After the exclusion of non-eligible publications (*n* = 27), ten studies (Table 1) were included in the meta-analysis. Several studies were based on the same population; namely two by de Greck and colleagues (de Greck et al., 2012, de Greck et al., 2011) as well as three publications by a working group from China (Song et al., 2015, Su et al., 2014, Zhang et al., 2015). We subsumed all of the results based on the sample within one dataset to prevent pseudo-replication and overestimating the influence of these individuals on the results. We analyzed seven datasets when using all studies, six when excluding structural studies. Thus, 243 individuals entered the full analysis including structural and functional studies, while 204 subjects the analysis focusing on functional studies (Table 1). Five foci were outside the mask in both analyses, whereby functional and structural analysis has a total of 107 foci and functional analysis 90 foci.

#### 3.1.1 ALE clusters of somatoform disorders

Five brain areas have been identified to repeatedly differ between patients with SD and healthy controls (Table 2) according to the calculated clusters. Out of the ten publications contributing to the calculations two did not contribute to any of the ALE clusters (Gündel et al., 2008, Yoshino et al., 2014).

#### 3.1.2 Functional areas of ALE clusters of SD in functional studies

In cluster 1 the dorsal posterior cingulate cortex (dPCC, Brodmann area (BA) 30 & 31) was identified to be significantly different across studies comparing patients and HC (Egloff et al., 2009, Song et al., 2015, Su et al., 2014, Zhang et al., 2015), while in Cluster 2 (de Greck et al., 2011, de Greck et al., 2012, Stoeter et al., 2007) the anterior prefrontal cortex (aPFC, BA 10) and in cluster 3 (Egloff et al., 2009, Stoeter et al., 2007) the insula (BA 13).

#### 3.1.3 Functional areas of ALE clusters of SD in structural and functional studies

**Fig. 2:**
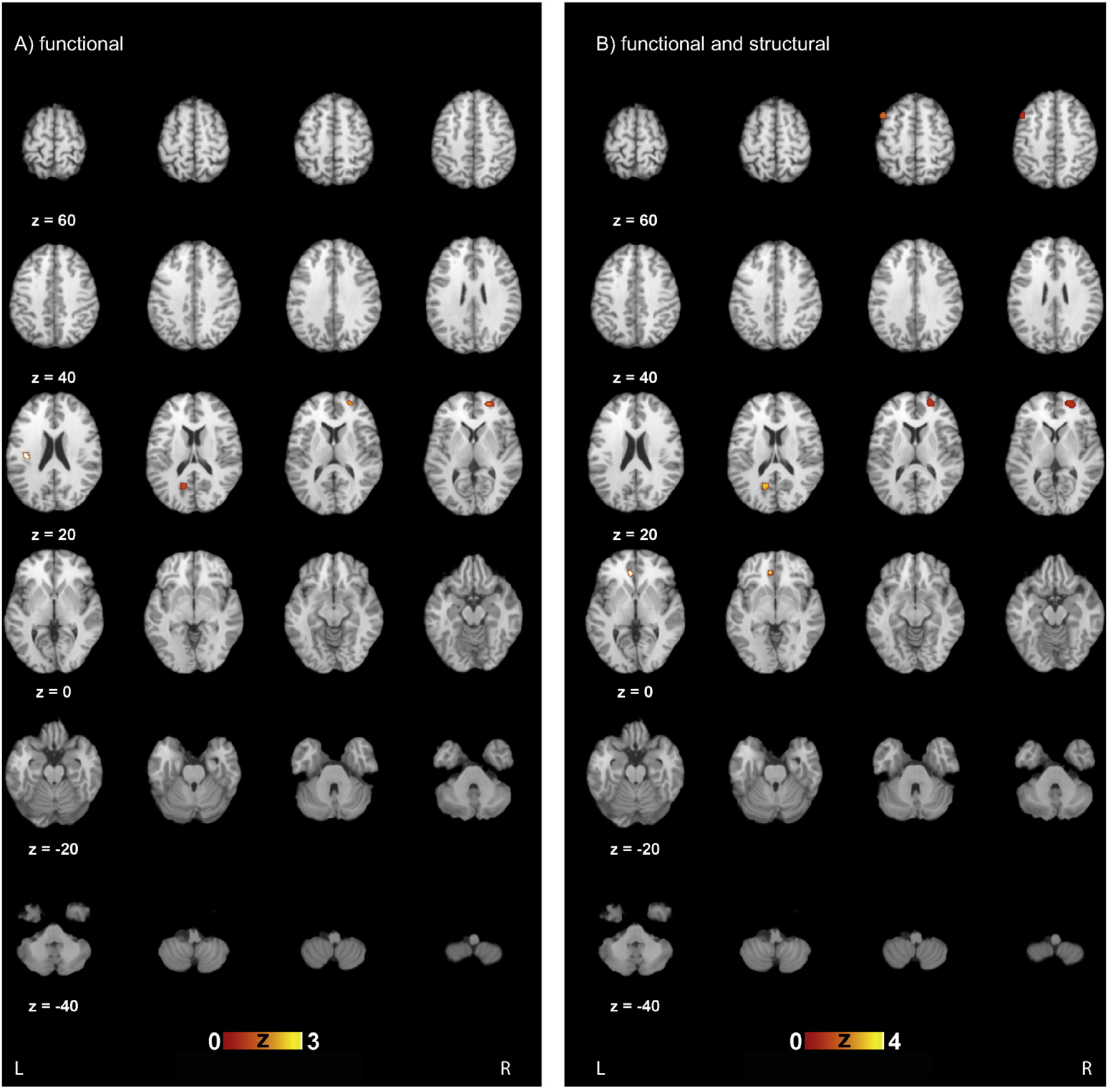
Results of the activation likelihood estimation (ALE) of A) functional studies and B) of functional and structural scans. Five areas were identified that were consistently differential in the comparison between healthy controls and patients with pain-related somatoform disorder (the premotor and supplementary motor cortexes, anterior prefrontal cortex, anterior cingulate cortex, insula, dorsal posterior cingulate cortex). Differential areas in A) functional analysis are dorsal posterior cingulate cortex, anterior prefrontal cortex, and insula and in B) functional and structural analysis the anterior prefrontal cortex, premotor and supplementary motor cortex (including lateral and medial supplementary cortex), dorsal posterior cingulate cortex, anterior cingulate cortex. Z coordinates in Talairach space are reported. Scale bar shows *z*-scores of ALE statistics with increasing significance from left to right. Only significant clusters are indicated with a cluster level above 0.05 and a *p*-value below 0.001.

In cluster 1 the aPFC (BA 10) was identified to be significantly different between patients and HC across several studies (de Greck et al., 2011, de Greck et al., 2012, Song et al., 2015, Stoeter et al., 2007, Su et al., 2014, Zhang et al., 2015). The premotor and supplementary motor cortex (PSMC, BA 6) including the lateral and medial supplementary motor area (BA 8), which is significant in cluster 2 (de Greck et al., 2011, de Greck et al., 2012, Egloff et al., 2009), is also within the  5 mm boundary in SD. While in cluster 3 (Egloff et al., 2009, Song et al., 2015, Su et al., 2014, Zhang et al., 2015) the dPCC (BA 30 & 31) is differentially activated. In cluster 4 the anterior cingulate cortex (ACC, BA 24 & 32) (Egloff et al., 2009, Valet et al., 2009) varies significantly between patients with SD and healthy controls (Fig. 2).

## 4 Discussion

In this meta-analysis, we present the results of an ALE-analysis on SD using the results of published neuro-imaging (MRI, SPECT, PET) studies. We herewith provide a first attempt to combine various studies to find consistent significant neurobiological differences between healthy controls and patients with SD. In both analyses combined we identified five areas showing functional and/or structural differences between the two groups of interest, namely the dorsal posterior cingulate cortex, anterior cingulate cortex, the anterior prefrontal cortex, the insula and the premotor and supplementary motor cortex.

We found significant differences between patients and controls in the ACC (Egloff et al., 2009, Valet et al., 2009). Functional studies showed that the ACC processes amongst others emotion (Kawamoto et al., 2012, Killgore and Yurgelun-Todd, 2007), attention (Nebel et al., 2005), and pain (Büchel et al., 2002, Lloyd et al., 2004). Changes in the ACC have been reported in cases of pain-induced depression (Barthas et al., 2014) and anxiety disorder (Shinoura et al., 2013). Patients with FSS experience alterations of attention, anticipation, and pain memories that correlate with increased activity of the ACC. These also correlate with the prefrontal areas and the ACC when stimulated below pain thresholds in both patients with FMS (Cook et al., 2004, Peyron et al., 2000) and IBS (Andresen et al., 2005, Naliboff et al., 2001). Additionally, studies on FMS repeatedly report reduced activation of the ACC during noxious stimulation (e.g.: Gracely et al., 2002, Jensen et al., 2009, Lee et al., 2013). The interaction of the ACC with prefrontal areas is in correlation with the negative forecasting while catastrophizing (Seminowicz and Davis, 2006).

The changed interaction between ACC and prefrontal areas might be represented in our data, as we also found changes in the aPFC, a part of the middle frontal gyrus (de Greck et al., 2011, de Greck et al., 2012, Stoeter et al., 2007). Chronic back pain disrupts normal functioning in the default mode network (DMN) also represented in the middle frontal gyrus of our results (Laird et al., 2009, Tagliazucchi et al., 2010). Changes in cortical thickness in the middle frontal gyrus have been shown to be reversible with back pain surgery (Seminowicz et al., 2011), whereby the resulting increases in gray matter in the MFG resulted in a reduction of pain and physical disability (Seminowicz et al., 2011). Patients with IBS show reductions of gray matter (Seminowicz et al., 2010) similar to the reductions of gray matter in the MFG in patients with SD, as well as reductions of activation in the middle frontal gyrus (de Greck et al., 2011, de Greck et al., 2012, Song et al., 2006, Stoeter et al., 2007).

Another important area for the occurrence of SD and that shares important interactions with the ACC is the insula. The insula is known to be important for pain processing (Fitzek et al., 2004, Kong et al., 2006, Schoedel et al., 2008, Strigo et al., 2003) and in paradigms when painful stimulation is expected (Sawamoto et al., 2000). Activation of the insula has been reported primarily for cutaneous pain rather than visceral pain (Strigo et al., 2003). In patients with FMS, the activation of the insula was connected to experiments on pain expectation (Gracely et al., 2002, Kim et al., 2013). Egloff et al. (2009) show that in cases of non-dermatomal somatosensory deficits in patients with chronic pain, the posterior part of the insula shows hypo-metabolic activation. This is especially significant as the area is essential for vibro-tactile and temperature discrimination tasks (Brooks et al., 2005). Contrary to the findings of Egloff et al. (2009); Stoeter et al. (2007) describe an increased activation of the insula as a result of the first application of pain in patients in comparison to healthy controls. The lateral nociceptive system, of which the insula is a part, indicated consistent hyper-perfusion, whereas the ACC and the more affective-motivational system did not (Stoeter et al., 2007). The increased activation in the insula may be correlated with the exaggerated expectancy of pain and attention in SD patients (Stoeter et al., 2007). This might be correlated with the anterior part of the insula, which is associated with cognitive-affective aspects of pain (Craig, 2003b). In FSS patients, the increased insular activation is correlated with heightened sensory coding of stimuli, which are coded as innocuous in healthy patients (Cook et al., 2004, Peyron et al., 2000). This effect has also been shown in patients with IBS (Berman et al., 2008). Medial and posterior parts of the insula are hypothesized to be involved in these somatosensory discriminative abilities (Craig, 2003b). It is hypothesized that an increased interoception is at work and is mediated via missing inhibitory descending prefrontal input and/or continued ascending arousal (Berman et al., 2008). The insula specifically the posterior part does receive somatosensory information of the thalamus and was brought into connection for a neural circuit model of somatosensory amplification (Perez et al., 2015). This circuit model proposes the thalamus to play an important part in SD (Perez et al., 2015). While the thalamus was not significantly different in our meta-analysis it shares many connections as important relaying area with areas that we found to be significantly changed in SD patients like the insula, cingulate cortex, prefrontal areas, and motor areas. Of the motor network we found the PSMC, which shares afferent and efferent connections with the thalamus, to be significantly changed across studies about SD.

Changes in the PSMC have previously been described in patients with chronic somatoform pain (Noll-Hussong et al., 2010). These changes often correlate with chronic pain because patients reduce movement in order to avoid painful stimulation (Rodriguez-Raecke et al., 2013). This explanation is supported by the increase of gray matter in correlation with time and improvement of motor function found in patients following a successful treatment for chronic pain, (Rodriguez-Raecke et al., 2013, Seminowicz et al., 2011). Outside changes in the motor cortex due to general chronic pain, alterations in the motor cortex are documented for patients with pain syndromes (Flor et al., 2001, Maihöfner et al., 2007, Swart et al., 2009). FSSs are related to modifications in the PSMC. IBS patients show urge-related differences in the PSMC (Kwan et al., 2005). Additionally, patients with fibromyalgia-syndrome show a reduction in the gray matter in the motor cortex (Puri et al., 2010).

Another significantly different area that is connected with the thalamus but also frontal brain areas like the BA 10 is the PCC (Maddock, 1999, Vann et al., 2009). The PCC is found to correlate with functional or structural changes in patients with SD. Increased activation in BA 23 correlates negatively with BA 30 activation, and BA 30 has a positive correlation with BA 31 (Cauda et al., 2010). In IBS and FMS, the PCC (BA 23, BA 31) and agranular retrolimbic area (ARA) differ between HC and patients (Boeckle et al., 2016). As PCC and ARA seem to be important parts of the DMN (Vann et al., 2009), a function already consistently found in 2-week-old infants (Gao et al., 2009), changes in DMN might underlie SD in general. Hyper-activation and hyper-connectivity in the DMN in schizophrenia are related to overtly increased self-reference, attention deficits, and impairments of working memory, while in depressive patients hyper-activation of the DMN might correlate with negative rumination (Whitfield-Gabrieli and Ford, 2012). Out of the four core regions (Whitfield-Gabrieli and Ford, 2012) of the DMN (medial prefrontal cortex (BA 10, 24, 23); PCC and retrosplenial cortex (BA 29, 30, 23, 13); left and right inferior parietal lobules (BA 39, 40)) all except the inferior parietal lobules seem to be dysfunctional in patients with SD. The DMN is also related to personal introspection, autobiographical memories, and thoughts about the future. Thus, disruptions of the DMN can lead to alexithymia as well as increased introspection and reflections of others (Buckner et al., 2008, Saxe et al., 2004, Whitfield-Gabrieli and Ford, 2012), aspects often observed in patients with SD.

In our analysis we found that evaluative regional areas like PCC, ACC, and insula are likely to have a high influence on the occurrence of SD. Thus it might be similar to sleep deprived individuals, who show an increased pain sensitivity (Schrimpf et al., 2015) that is based on changes of the evaluative brain network, which influences clinical pain complaints but not pain thresholds (Busch et al., 2012). Therefore, it seems that central mechanisms like central sensitization might have a higher impact than peripheral mechanisms like spinal sensitization. This unspecific network of higher brain functions was also called ‘neuromatrix’ (Melzack, 2001). This network that previously was also called ‘painmatrix’ (Iannetti and Mouraux, 2010) is not specific for pain as it is active in various conscious processes (Melzack, 2001). The ‘neuromatrix’ has been divided in three subsystems, namely the lateral (sensory and discriminative), medial (affective and motivational) and frontal (cognitive and evaluative) system (Apkarian et al., 2005, Melzack, 2001). SD patients appear to have mainly changes in medial and frontal parts of the neuromatrix, responsible for affective and evaluative responses. This has specifically shown for pain where affective components were processed in dACC, while sensory components are processed by primary and secondary somatosensory cortex (S1, S2) and the posterior part of the insula (PI) (Treede et al., 1999). According to our meta-analysis the somatosensory cortex is not found to be different between SD patients and HC in our study. Though, differences in the posterior part of the insula, responsible for somatosensory processes of pain, do show differences.

### 4.1 Limitations

By undertaking an ALE analysis of SD, we were able to include a sample of 243 (204 functional) individuals from ten respectively 8 functional studies, and nine (7 functional) datasets. In our analysis two studies did not contribute to any of the resulting clusters (Gündel et al., 2008, Yoshino et al., 2014). This resulted in a sample, with which we were able to identify neurobiological similarities within this disorder. We have to report, that the foci included in our study are uncorrected for intervening factors, such as varying experimental design, imaging parameters, analysis software, etc. Still, we think that due to the meta-analytic approach, it was possible to identify some commonalities amongst patients via this ALE analysis. Especially when comparing our results with previous theoretical models based on various imaging studies across paradigms and neuroimaging methods, our meta-analytical perspective bases its conclusion on statistical testing and can thus contrast to merely theoretically driven functional models. Still, as some of the studies that were included for the proposal of the circuit model did not report any coordinates and were thus not included in our analysis, some differences between the model of Perez et al. (2015) and ours might be based on the difference between inclusion criteria. Additionally, most studies used mainly female participants. Thus, all results may be applicable to female patients with SD only. In future studies, it would be highly advantageous to look for studies including male patients with SD, as it has been shown that differences between male and female patients do exist in somatoform disorders (Labus et al., 2008, Labus et al., 2013). Finally, specificity of our results for SD might be limited, as Hsu et al. (2009) has shown that gray matter changes in FMS patients are dependent on affective disorder rather than on FMS. A similar effect might underlie the selected studies. This might be especially critical, as it has been found that SD and depression are highly comorbid (Busch et al., 2012). Thus, future functional and structural studies should control for affective disorders. Additionally, a recent meta-analysis revealed that changes in the dorsal ACC, as well as the left and right insula are common to all psychiatric diagnoses (Goodkind et al., 2015) and might not be specific to SD.

## 5 Conclusions

This meta-analysis underlines neurobiological aspects of SD. In SD, the premotor and supplementary motor cortex, the middle frontal gyrus, the anterior cingulate cortex, the insula and the posterior cingulate cortex seem to be of particular importance. Furthermore, these results seem in line with the hypothesis of “central sensitization” (CS) (Bourke et al., 2015) especially when looking at the neurobiological model (Perez et al., 2015). Still discrepancies do exist and future research is needed in order to better understand the underlying brain network and mechanisms. However, it is important to also consider the influence of a co-morbid depression. Hsu et al. (2009), for example, found no consistent difference in gray matter volume between FMS patients when controlling for affective disorder (Hsu et al., 2009). Thus, future neuroimaging studies should consider the role of affective disorders when investigating somatoform disorder. Still, it is conceivable that many FSS, independent of the clinical impression, show similar neurobiological processes. However, we expect specificities to be at work in each single FSS and thus presented results for SD only. Investigating neurobiological mechanisms of various FSS like SD will help to understand the aetiology of the disorders, lead away from medically unexplained syndromes, and might in the long run help to develop better treatment programs for patients suffering from SD.

## Conflict of interests

The authors have no conflicts of interest to report.

## Figures and Tables

**Table 1:** Studies included in the analysis. Type of disorder as indicated in the original publication.

Citation	Type of disorder	Number of participants	Imaging method	Task
de Greck et al. (2011)	Somatoform disorder	P:20 (12f); HC: 20 (12f)	MRI (1.5T)	Monetary reward task
de Greck et al. (2012)	Somatoform disorder	P:20 (12f); HC: 20 (12f)	MRI (1.5T)	Emotional empathy
Gündel et al. (2008)	Somatoform pain disorder	P:12 (12f); HC: 20 (13f)	MRI (1.5T)	Noxious heat stimuli
Egloff et al. (2009)	Chronic pain, not explained by peripheral tissue damage	P: 11 (6f); HC: 12 (6f)	PET	Glucose metabolism resting state
Song et al. (2015)	Somatization disorder	P: 25 (21f); HC: 28 (22)	MRI (3.0T)	Resting state (ReHo)
Stoeter et al. (2007)	Somatoform pain disorder	P:17 (11f); HC: 17 (11f)	MRI (1.5T)	Pin-prick stimulation, emotional and cognitive stress
Su et al. (2014)	Somatization disorder	P: 25 (21f); HC: 28 (22)	MRI (3.0T)	Resting state (fALFF)
Valet et al. (2009)	Pain disorder, without any somatic cause	P:14 (14f); HC: 14 (14f)	MRI (1.5T)	VBM structural scan
Yoshino et al. (2014)	Somatoform pain disorder	P: 9 (4); HC: 20 (13)	MRI (1.5T)	Resting state (ReHo)
Zhang et al. (2015)	Somatization disorder	P: 25 (21f); HC: 28 (22)	MRI (3.0T)	DTI structural scan

P: patients; HC: healthy controls; T: Tesla; fMRI: magnetic resonance tomography; PET: positron emission tomography; f: number of females; ReHo: regional homogeneity; fALFF: fractional amplitude of low-frequency fluctuations.

**Table 2:** ALE clusters.

	Cluster #	# of datasets	Gray matter at center	Additional gray matter within 5 mm	Center	Vol. mm^3^	Studies included
Functional studies	Cluster 1	2	BA 31: dorsal posterior CC	BA 30: part of posterior CC	x = − 13.2 y = − 62.6 z = 15.1	384	Egloff et al. (2009), Song et al. (2015), Su et al. (2014), Zhang et al. (2015)
Cluster 2	2	BA 10: anterior prefrontal cortex		x = 23.4 y = 50.3 z = 7.3	304	de Greck et al., 2011, de Greck et al., 2012, Stoeter et al. (2007)
Cluster 3	2	BA 13: Insula		x = − 37.3 y = − 21.1 z = 19.8	256	Egloff et al. (2009), Stoeter et al. (2007)
Structural & functional studies	Cluster 1	3	BA 10: anterior prefrontal cortex		x = 21.8 y = 50.9 z = 7.5	808	de Greck et al., 2011, de Greck et al., 2012, Song et al. (2015), Stoeter et al. (2007), Su et al. (2014)Zhang et al. (2015)
Cluster 2	2	BA 6: premotor and supplementary motor cortex	BA 8: lateral and medial supplementary motor area	x = − 43.2 y = 14.6 z = 48.7	376	de Greck et al., 2011, de Greck et al., 2012, Egloff et al. (2009)
Cluster 3	2	BA 31: dorsal posterior CC	BA 30: part of posterior cingulate cortex	x = 13.3 y = − 62.5 z = 15.1	352	Egloff et al. (2009), Song et al. (2015), Su et al. (2014), Zhang et al. (2015)
Cluster 4	2	BA 32: anterior CC	BA 24: part of anterior CC	x = − 6.2 y = 33.7 z = − 2.2	272	Egloff et al. (2009), Valet et al. (2009)

Brodmann Area (BA), Cingutale cortex (CC), number (#), x-, y-, *z*-coordinates in Talairach space.
